# The effect of omega-3 supplementation on metabolic, inflammatory and oxidative stress biomarkers in pregnant women: a systematic review and meta-analysis

**DOI:** 10.3389/fnut.2025.1639906

**Published:** 2025-09-22

**Authors:** Mohamed J. Saadh, Zahraa Sabah Ghnim, Morug Salih Mahdi, Lalji Baldaniya, Sairah Abdul Karim, Manish Srivastava, Deepak Bhanot, Jasur Rizaev, Waam Mohammed Taher, Mariem Alwan, Mahmood Jasem Jawad, Atheer Khdyair Hamad

**Affiliations:** ^1^Faculty of Pharmacy, Middle East University, Amman, Jordan; ^2^College of Pharmacy, Alnoor University, Nineveh, Iraq; ^3^College of MLT, Ahl Al Bayt University, Karbala, Iraq; ^4^Marwadi University Research Center, Department of Pharmaceutical Sciences, Faculty of Health Sciences, Marwadi University, Rajkot, Gujarat, India; ^5^Management and Science University, Shah Alam, Selangor, Malaysia; ^6^Department of Endocrinology, National Institute of Medical Sciences, NIMS University Rajasthan, Jaipur, India; ^7^Centre for Research Impact and Outcome, Chitkara University Institute of Engineering and Technology, Chitkara University, Rajpura, Punjab, India; ^8^Department of Public Health and Healthcare Management, Samarkand State Medical University, Samarkand, Uzbekistan; ^9^College of Nursing, National University of Science and Technology, Nasiriyah, Iraq; ^10^Pharmacy College, Al-Farahidi University, Baghdad, Iraq; ^11^Department of Pharmacy, Al-Zahrawi University College, Karbala, Iraq; ^12^Gilgamesh Ahliya University, Baghdad, Iraq

**Keywords:** omega-3, pregnancy, lipid, inflammation, oxidative stress

## Abstract

**Background:**

This meta-analysis evaluates the effects of omega-3 supplementation on metabolic, inflammatory, and oxidative stress in pregnancy women by synthesizing findings from randomized controlled trials (RCTs), as existing evidence remains inconclusive.

**Methods:**

A systematic search was conducted using PubMed, Scopus, and Web of Science until July 2024. Random-effects models were applied to estimate each outcome's standardized mean difference (SMD) and 95% confidence intervals (CI).

**Results:**

A total of 14 studies were included in the meta-analysis. The duration of omega-3 supplementation ranged from 6 to 29 weeks. Omega-3 supplementation did not have a significant effect on FBS (SMD = −0.74, 95% CI: −1.94, 0.45), and insulin (SMD = −0.76, 95% CI: −1.77, 0.24), TC (SMD = 0.11, 95% CI: −0.20, 0.42), and LDL-C (SMD = 0.32, 95% CI: −0.17, 0.81), IL-6 (SMD = 2.12, 95% CI: −0.56, 4.80), MDA (SMD = −1.67, 95% CI: −3.39, 0.05), and TAC (SMD = 2.59, 95% CI: −0.37, 5.54). However, triglyceride (SMD = −0.96, 95% CI: −1.77, −0.16) and CRP (SMD = −0.98, 95% CI: −1.86, −0.11) significantly decreased, and HDL-C cholesterol levels significantly increased (SMD = 0.72, 95% CI: 0.21, 1.22) following omega-3 supplementation.

**Conclusion:**

This study suggests omega-3 supplementation may improve lipid profiles and reduce inflammatory biomarkers during pregnancy. However, the presence of heterogeneity across trials highlights the need for further well-conducted studies. Thus, findings should be interpreted with caution.

## Introduction

Pregnant women undergo substantial metabolic adaptations—progressive insulin resistance, dyslipidemia, heightened inflammation, and oxidative stress—particularly pronounced in gestational diabetes mellitus (GDM), elevating risks of macrosomia, preeclampsia, and long-term metabolic sequelae in both mother and offspring (1). Nutritional strategies that target these disturbances are of significant clinical interest. Omega-3 polyunsaturated fatty acids (PUFAs), notably EPA and DHA, exhibit insulin-sensitizing, lipid-regulating, anti-inflammatory, and antioxidant properties in general populations with type 2 diabetes and metabolic syndrome (2–4).

Although existing RCT evidence in pregnant populations is limited, a focused systematic review and meta-analysis of trials up to 2019 demonstrated that omega-3 supplementation significantly improved fasting plasma glucose, HOMA-IR, and reduced high-sensitivity C-reactive protein (hs-CRP) in women with GDM, while effects on nitric oxide, lipid fractions, IL-6, and malondialdehyde were not significant (5). Another meta-analysis also found reductions in fasting insulin, triglycerides, VLDL-cholesterol, modest HDL-cholesterol increases, and CRP declines (6). RCTs involving omega-3 plus vitamin E co-supplementation in GDM women also report significant increases in total antioxidant capacity (TAC), nitric oxide (NO), and reductions in malondialdehyde (MDA). However, effects on glutathione were inconsistent (7). A network meta-analysis further confirmed that omega-3 with vitamin E outperformed placebo in improving oxidative stress markers (TAC and MDA) in GDM pregnancies (8–10). In summary, while individual trials yield heterogeneous findings, pooled data suggest that omega-3 supplementation—especially when combined with antioxidants—can beneficially modulate glycemic indices, lipid metabolism, inflammation, and redox balance in pregnant women with GDM. Yet, dosage, intervention duration, and outcome selection inconsistencies highlight the imperative for a comprehensive systematic review and meta-analysis. Accordingly, this study aims to quantify the effects of omega-3 PUFAs on glycemic, lipid, inflammatory, and oxidative stress biomarkers in pregnant women, providing evidence-informed insights for clinical nutrition guidelines.

## Materials and methods

This study was performed following the PRISMA (Preferred Reporting Items of Systematic Reviews and Meta-Analysis) statement guidelines (11). The protocol was registered in the International Prospective Register of Systematic Reviews (PROSPERO) database.

### Search strategy

A thorough literature review was conducted using Web of Science, Scopus, and PubMed, including all records up to July 2024. The search terms included various combinations of keywords related to pregnancy, gestation, omega-3 fatty acids (e.g., Omega-3 Fatty Acid, n-3 Oil, n-3 Fatty Acids, n-3 Polyunsaturated Fatty Acids, Omega-3 Polyunsaturated Fatty Acid), and metabolic outcomes (e.g., lipid, total cholesterol (TC), high-density lipoprotein cholesterol [HDL-C], low-density lipoprotein cholesterol [LDL-C], triglycerides [TG], fasting blood glucose, inflammation, and oxidative stress) and intervention, controlled trial, randomized controlled trial, clinical trial, RCT (see Supplementary Table 1 for specific search terms). Additionally, we examined the reference lists of existing systematic reviews and meta-analyses to ensure comprehensive coverage. No language restrictions were applied. Our search methodology was based on the Cochrane PICO framework: (1) Participants—pregnant women at any stage of gestation or age; (2) Intervention—omega-3 supplementation; (3) Comparator –placebo, no intervention, or lower doses of the same supplement; and (4) Outcomes—metabolic and biochemical measures such as lipid profile (total cholesterol, HDL-C, LDL-C, triglycerides), fasting glucose, inflammatory markers, and oxidative stress indicators.

### Study selection

We included randomized controlled trials and other controlled study designs involving pregnant women of any age, body mass index, or pregnancy-related health conditions such as gestational diabetes, preeclampsia, or pregnancy-induced hypertension. The eligible interventions included omega-3 fatty acids (EPA, DHA, or ALA) alone or with common pregnancy supplements like vitamin E, D, or folic acid. Studies were excluded if they involved infants, children, or adolescents; were published in languages other than English; were animal or *in vitro* experiments; or were secondary sources such as systematic reviews, meta-analyses, editorials, conference abstracts, gray literature, or book chapters.

### Data extraction

Two separate researchers reviewed and selected studies to reduce bias and maintain uniformity. Disagreements were settled through discussion, with the principal investigator acting as a tiebreaker for unresolved issues—a standard approach in systematic reviews. Studies that appeared potentially relevant were obtained in full and evaluated independently by both researchers, with exclusion reasons recorded in line with PRISMA standards. When key outcome data were absent or inconsistently reported, we emailed the authors to obtain the needed information. If no response was received or the data remained inaccessible, the outcomes were omitted or marked as missing.

We gathered the specified data using standardized forms in all the studies included. This data encompassed the first author's name, publication year, location of the study, type of trial (either parallel or crossover), characteristics of participants such as gestational age and health condition, the number of participants, length of the intervention, the form and amount of omega-3 used, details on any additional supplements, and the outcomes measured—including glycemic, lipid, inflammatory, and oxidative stress markers. When feasible, two researchers independently extracted the data and verified it against each other's work to confirm its accuracy and completeness.

### Assessment of the quality of studies

Two reviewers independently assessed the methodological quality of the included randomized controlled trials. Any disagreements between reviewers were resolved through discussion until consensus was reached. The evaluation was conducted using the Cochrane Collaboration's Risk of Bias tool (RoB 2) (12).

### Quantitative data synthesis and statistical analysis

The mean outcome change was determined by subtracting the final follow-up measurement from the baseline. To compute the standard deviation of this mean change, the following formula was applied: SD = Square root of [(SD pre-treatment)^2^ + (SD post-treatment)^2^ – (2R × SD pre-treatment × SD post-treatment)], assuming a correlation coefficient (R) of 0.5 (13). Study heterogeneity was assessed using Cochran's *Q* test and *I*^2^ test, with values ≥50% (or *p* < 0.05) indicating significant heterogeneity, where the random effects model was applied for reporting (14). Subgroup analyses were performed based on various covariates to investigate possible reasons for heterogeneity across combined studies. When fewer than 10 studies reported outcomes, formal assessments for publication bias, such as funnel plots and Egger's test, were not conducted, following established standards, because these methods lack sufficient statistical power with small sample sizes (15). A meta-regression analysis examined how study-level covariates—such as maternal overweight or obesity, GDM or preeclampsia status, and the risk-of-bias score—might influence the effect estimates. A sensitivity analysis using the leave-one-out approach was conducted to evaluate how each study influences the overall effect size estimate (16). Statistical significance was defined as *P* < 0.05.

## Results

### Study selection process

**Figure 1:**
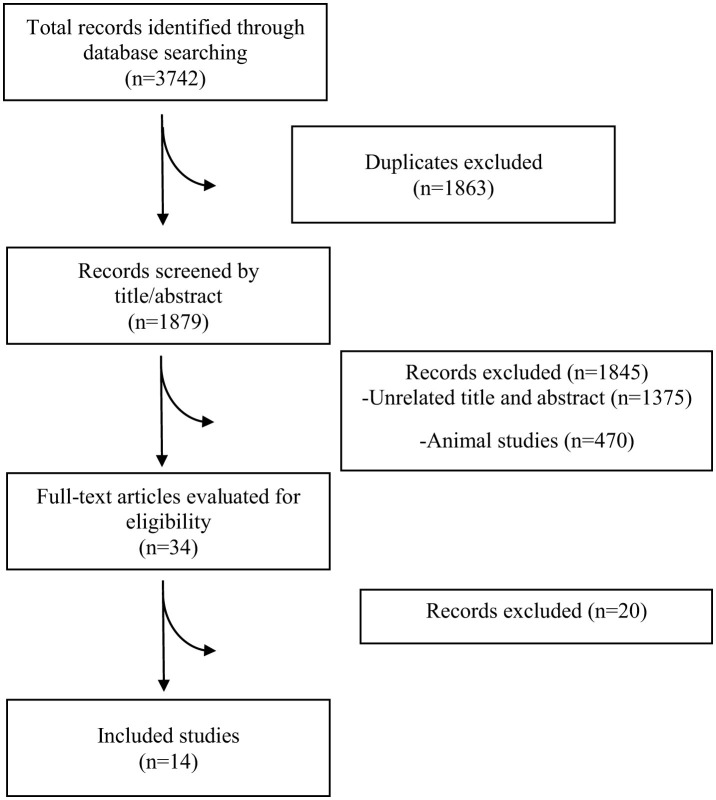
PRISMA flow diagram.

The study selection process is described in Figure 1, following the PRISMA 2020 standards for systematic reviews. A total of 3,742 records were initially identified. After removing 1,863 duplicates, 1,879 unique entries were subjected to title and abstract screening. Of these, 34 full-text articles were assessed for eligibility based on specific inclusion and exclusion criteria. In the end, 14 studies had enough data to be included in the meta-analysis (7, 9, 10, 17–27).

### Study characteristics

The 14 RCTs included in the meta-analysis were conducted across diverse geographic settings: seven in Iran; two in the USA; and one each in Chile, Finland, Germany, Norway, and Spain (Table 1). Participants were enrolled between 10 and 29 weeks of gestation, representing early and mid-pregnancy time points. Omega-3 supplementation doses ranged from 300 to 4,000 mg per day, administered as fish oil preparations (containing DHA, EPA, or both) or flaxseed oil formulations rich in ALA. The study populations encompassed healthy pregnant women, women with overweight or obesity, and those diagnosed with GDM. Supplementation durations varied substantially, spanning 6–29 weeks, and covered interventions initiated from early pregnancy through to late gestation.

**Table 1:** Summary of the studies.

**First author, Year**	**Design**	**Participants (treatment/control)**	**Place**	**Health condition**	**Gestational age (wk)**	**Duration (wk)**	**Treatment**	**Control**
Helland et al. 2006	RCT	160/154	Norway	Healthy	17–19	~17 weeks	10 ml/d cod liver oil (1,183 mg DHA + 803 mg EPA)	Corn oil (4,747 mg LA + 92 mg ALA per 10 mL)
Franke et al. 2010	DB-RCT	59/65	Germany	Healthy	22	~18	Milk-based supplement (500 mg DHA + 150 mg EPA)	Placebo
Samimi et al. 2015	DB-RCT	28/28	Iran	GDM	24–28	6	1,000 mg omega-3 (180 mg EPA + 120 mg DHA)	Placebo
Haghiac et al. 2015	DB-RCT	25/24	USA	Overweight/obesity	10–16	Until term	DHA (800 mg/day) + EPA (1,200 mg/day)	Placebo
Faraji et al. 2016	RCT	45/47	Iran	Healthy	16–20	~20	Fish oil (1,000 mg/day)	Placebo
Taghizadeh et al. 2016	DB-RCT	30/30	Iran	GDM	26	6	1,000 mg omega-3 (flaxseed oil) + 400 IU vitamin E	Placebo
Rodriguez-Santana et al. 2017	DB-RCT	24/22	Spain	Healthy	28	26.5	Fish oil drink (392 mg omega-3: 320 mg DHA + 72 mg EPA)	Non-supplemented dairy drink
Razavi et al. 2017	DB-RCT	30/30	Iran	GDM	24–28	6	1,000 mg omega-3 (180 mg EPA + 120 mg DHA twice daily)	Placebo
Vahedi et al. 2018	TB-RCT	75/75	Iran	Healthy	20	~18	1.2 g DHA + 1.8 g EPA	Placebo
Garmendia et al. 2018	RCT (2 × 2 factorial)	252/251	Chile	Overweight/obesity	< 15	Until delivery	Routine counseling + 800 mg/day DHA	Routine counseling + 200 mg/day DHA
Jamilian et al. 2018	DB-RCT	20/20	Iran	GDM	24–28	6	1,000 mg fish oil (180 mg EPA + 120 mg DHA twice/day)	Placebo
Jamilian et al. 2020	DB-RCT	26/25	Iran	GDM	24–28	6	2,000 mg/d flaxseed oil (800 mg α-linolenic acid)	Placebo
Valentine et al. 2021	RCT, multi-site, double-blind superiority	465/437	USA	Healthy	12–20	Until delivery	1,000 mg/day DHA	200 mg/day DHA
Houttu et al. 2021	DB-RCT	93/92	Finland	Overweight/obesity	13.8 ± 2.1	Early pregnancy to 6 months postpartum	Fish oil (1.9 g DHA + 0.22 g EPA)	Placebo

### Risk of bias assessment

Table 2 details the risk of bias evaluation for the included randomized controlled trials. Among the 14 RCTs included, most showed low risk of bias for randomization, deviation from intended interventions, missing outcome data and outcome measurement. However, selective reporting was a concern in 13 studies due to lack of existing protocols or pre-registration. In total, 13 trials were assessed as having some concerns due to reporting limitations, while one study was assessed as being at low risk of bias. No studies were classified as high risk, indicating generally strong methodology with minor concerns about transparency of reporting.

**Table 2:** Summarizes the Risk of Bias (RoB 2) assessment for the included randomized controlled trials.

**Study (Author, Year)**	**Randomization process**	**Deviations from intended interventions**	**Missing outcome data**	**Outcome measurement**	**Selective reporting**
Houttu et al. 2020	Low	Low	Low	Low	Some concerns
Valentine et al. 2021	Low	Low	Low	Low	Low
Faraji et al. 2016	Some concerns	Low	Some concerns	Low	Some concerns
Rodriguez-Santana et al. 2017	Low	Low	Low	Low	Some concerns
Garmendia et al. 2015	Some concerns	Low	Some concerns	Low	Some concerns
Taghizadeh et al. 2016	Low	Low	Low	Low	Some concerns
Razavi et al. 2017	Low	Low	Low	Low	Some concerns
Haghiac et al. 2015	Low	Low	Low	Low	Some concerns
Jamilian et al. 2017	Low	Low	Low	Low	Some concerns
Franke et al. 2010	Low	Low	Low	Low	Some concerns
Helland et al. 2006	Low	Low	Some concerns	Low	Some concerns
Jamilian et al. 2020	Low	Low	Low	Low	Some concerns
Vahedi et al. 2018	Low	Low	Low	Low	Some concerns
Samimi et al. 2015	Low	Low	Low	Low	Some concerns

### Effect of omega-3 supplementation on glycemic indices

**Figure 2:**
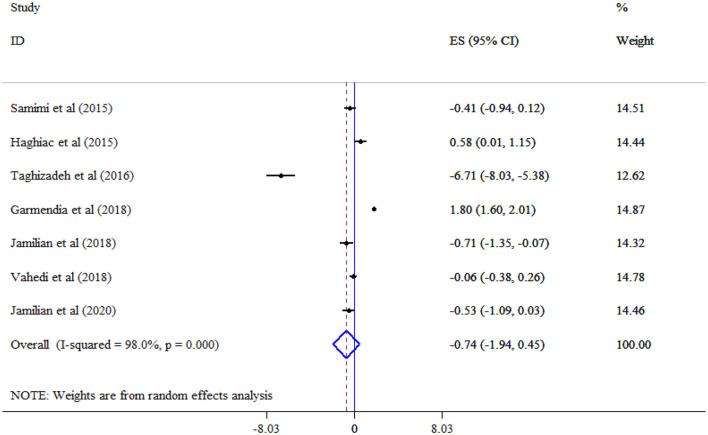
Effect of mega-3 supplementation FBS.

**Figure 3:**
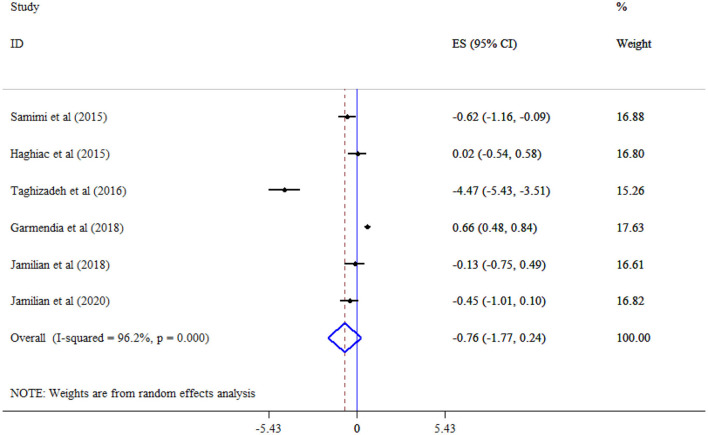
Effect of mega-3 supplementation insulin.

FBS (Seven studies, SMD = −0.74, 95% CI: −1.94, 0.45; *p* = 0.224; *I*^2^ = 98.0%, *p* < 0.001) (Figure 2), and insulin (Six studies, SMD = −0.76, 95% CI: −1.77, 0.24; *p* = 0.136; *I*^2^ = 96.2%, *p* < 0.001) (Figure 3) level did not significantly decrease following omega-3 supplementation with substantial heterogeneity. Subgroup analysis revealed the most substantial effects in patients with GDM and gestational age >20 weeks (Table 3).

**Table 3:** Subgroup analysis.

	**Number**	**SMD (95% CI)**	***I*^2^ (%)**
**Omega-3 on FBS**			
Overall	7	−0.74 (−1.94, 0.45)	98.0
**Gestational age (week)**			
≤ 20	3	0.78 (−0.55, 2.11)	97.9
>20	4	−1.96 (−3.68, −0.23)	96.2
**Intervention duration (week)**			
< 15	5	−1.22 (−3.09, 0.65)	97.0
≥15	2	0.21 (−0.41, 0.84)	0.0
**Health condition**			
GDM	4	−1.96 (−3.68, −0.23)	96.2
Obesity	2	1.22 (0.03, 2.41)	93.6
Healthy	1	−0.06 (−0.38, 0.26)	–
**Sample size**			
< 60	4	−0.26 (−0.82, 0.30)	73.8
≥60	3	−1.48 (−3.79, 0.82)	99.1
**Omega-3 on insulin**			
Overall	6	−0.76 (−1.77, 0.24)	96.2
**Gestational age (week)**			
≤ 20	2	0.40 (−0.22, 1.01)	78.0
>20	4	−1.37 (−2.82, 0.08)	95.2
**Intervention duration (week)**			
< 15	5	−0.94 (−2.17, 0.30)	96.9
≥15	1	0.02 (−0.54, 0.58)	–
**Health condition**			
GDM	4	−1.37 (−2.82, 0.08)	95.2
Obesity	2	0.40 (−0.22, 1.01)	78.0
**Sample size**			
< 60	4	−0.31 (−0.60, −0.02)	6.0
Overall	8	−0.96 (−1.77, −0.16)	97.2
**Gestational age (Week)**			
≤ 20	3	−0.93 (−2.31, 0.44)	98.7
>20	5	−0.98 (−1.98, 0.03)	94.1
**Intervention duration (week)**			
< 15	5	−1.45 (−2.64, −0.26)	96.5
≥15	3	−0.21 (−0.80, 0.38)	89.9
**Health condition**			
GDM	4	−1.23 (−2.61, 0.15)	94.8
Obesity	1	−2.29 (−2.51, −2.06)	–
Healthy	3	−0.21 (−0.80, 0.38)	89.9
**Sample size**			
< 60	3	−0.42 (−1.00, 0.15)	66.9
≥60	5	−1.27 (−2.37, −0.18)	98.2

### Effect of omega-3 supplementation on lipid profile

**Figure 4:**
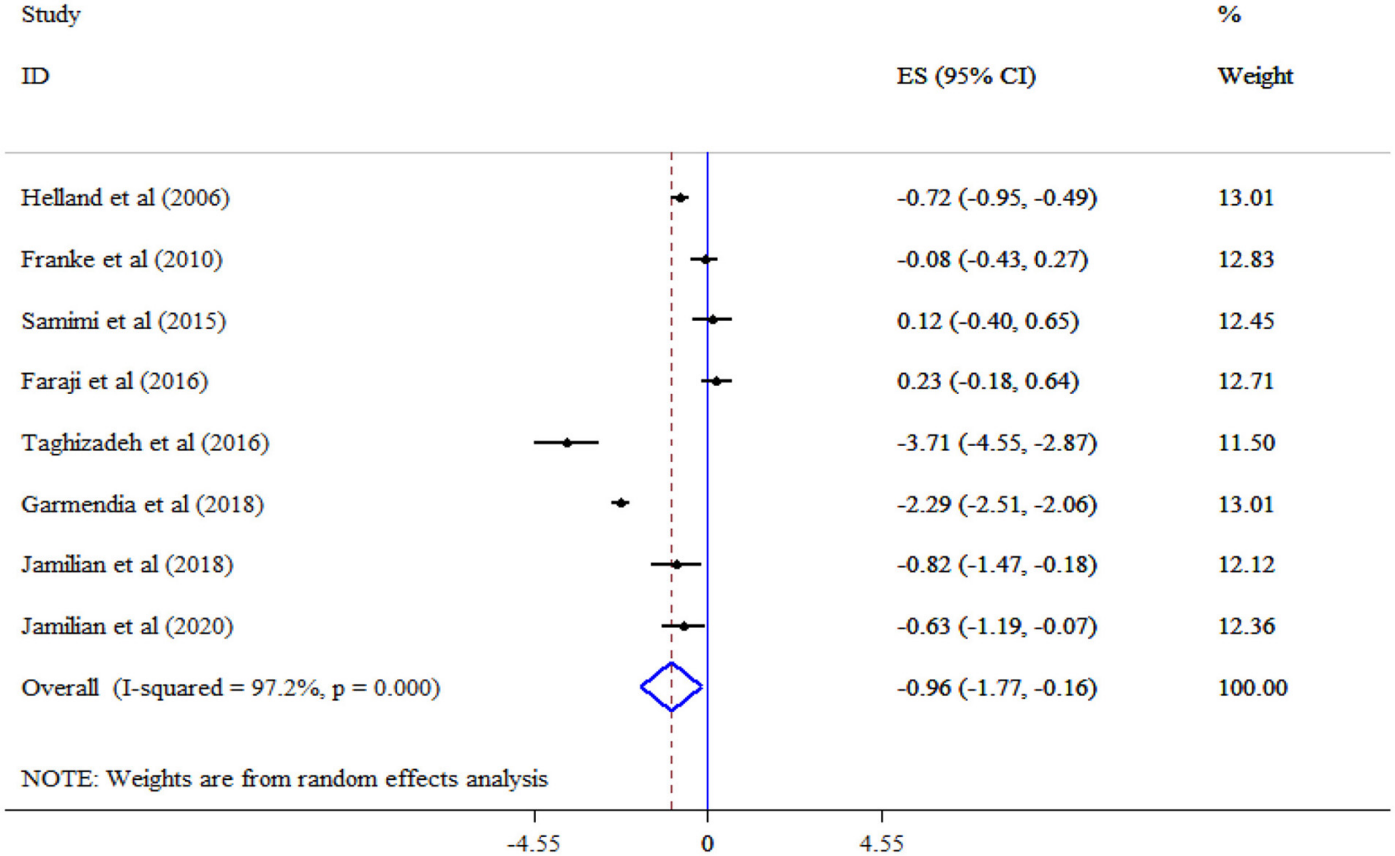
Effect of mega-3 supplementation TG.

**Figure 5:**
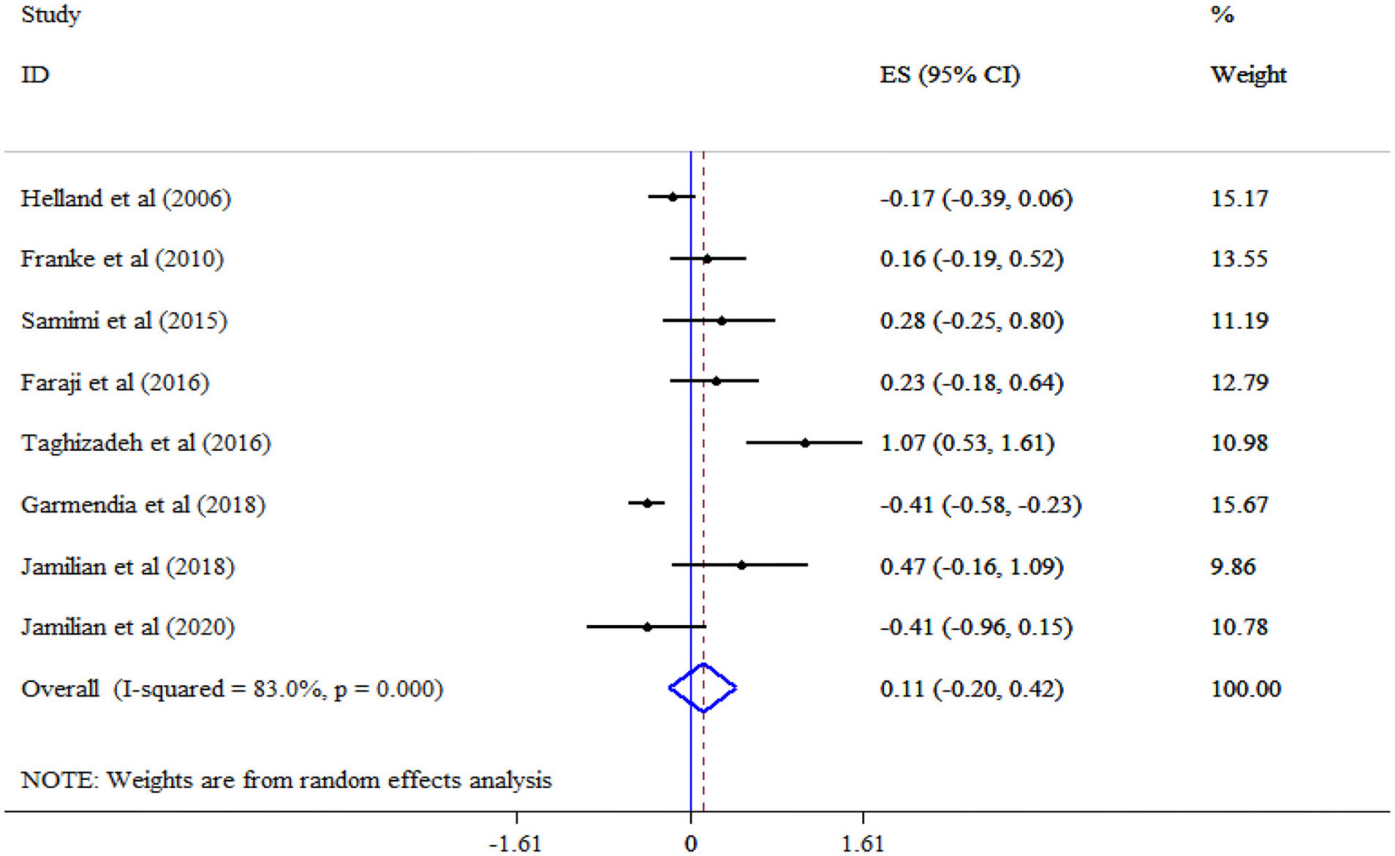
Effect of mega-3 supplementation TC.

**Figure 6:**
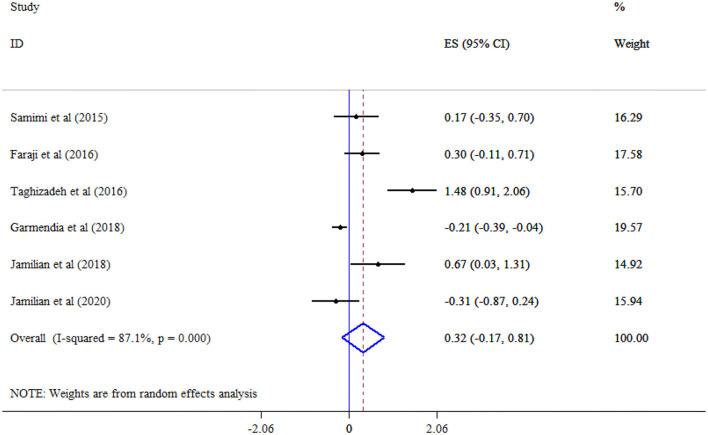
Effect of mega-3 supplementation LDL-C.

**Figure 7:**
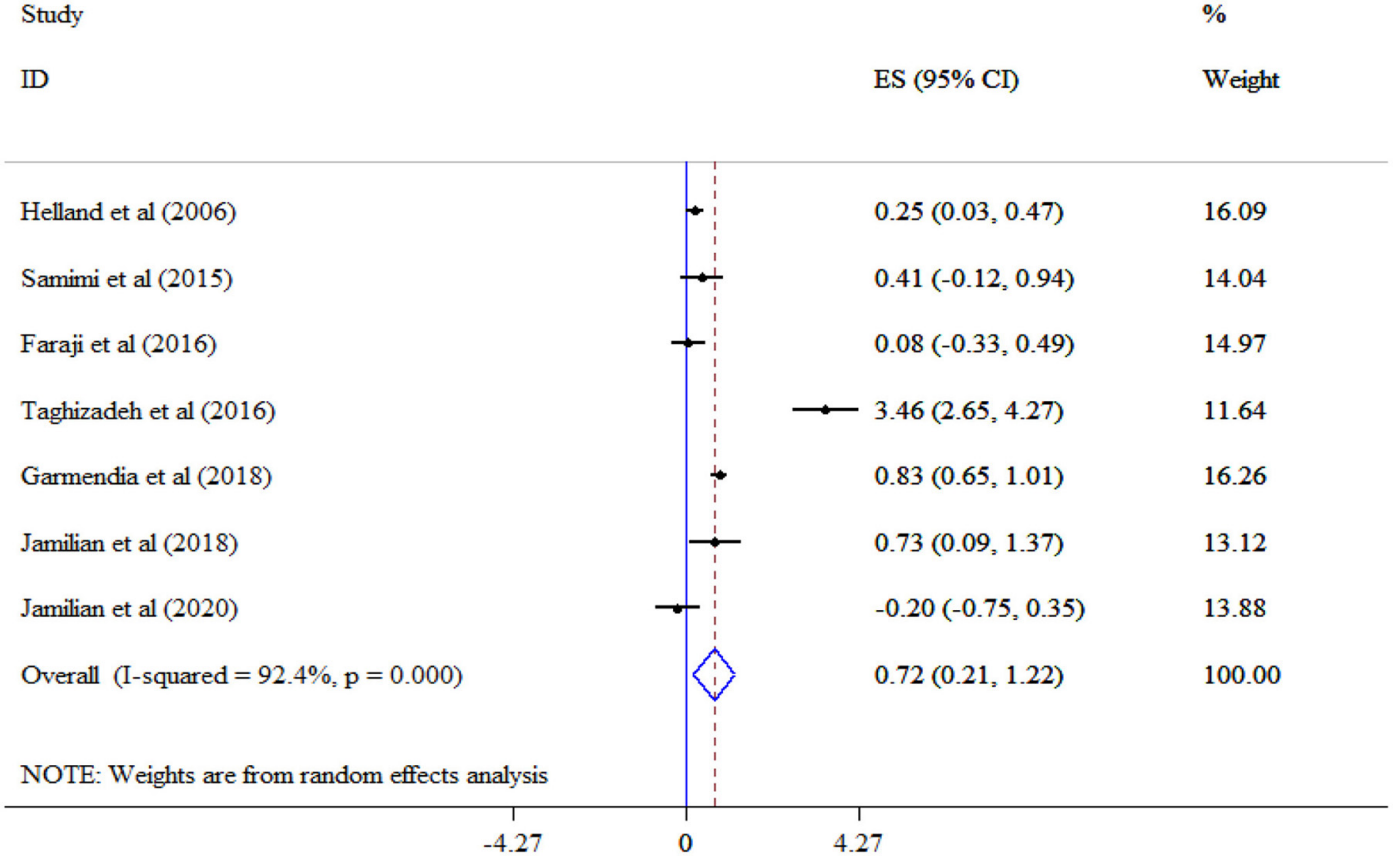
Effect of mega-3 supplementation HDL-C.

The analysis revealed that omega-3 supplementation significantly improved TG (Eight studies, SMD = −0.96, 95% CI: −1.77, −0.16; *p* = 0.019; *I*^2^ = 97.2%, *p* < 0.001) (Figure 4), but not TC (Eight studies, SMD = 0.11, 95% CI: −0.20, 0.42; *p* = 0.478; *I*^2^ = 83.0%, *p* < 0.001) (Figure 5), and LDL-C (Six studies, SMD = 0.32, 95% CI: −0.17, 0.81; *p* = 0.195; *I*^2^ = 87.1%, *p* < 0.001) (Figure 6) levels. A significant increase in HDL-C (Seven studies, SMD = 0.72, 95% CI: 0.21, 1.22; *p* = 0.006; *I*^2^ = 92.4%, *p* < 0.001) levels was observed following omega-3 supplementation (Figure 7). Subgroup analysis revealed the most substantial effects in patients with GDM, in a sample size of ≥60 and intervention duration < 15 weeks (Table 3).

### Effect of omega-3 supplementation on inflammatory biomarkers

**Figure 8:**
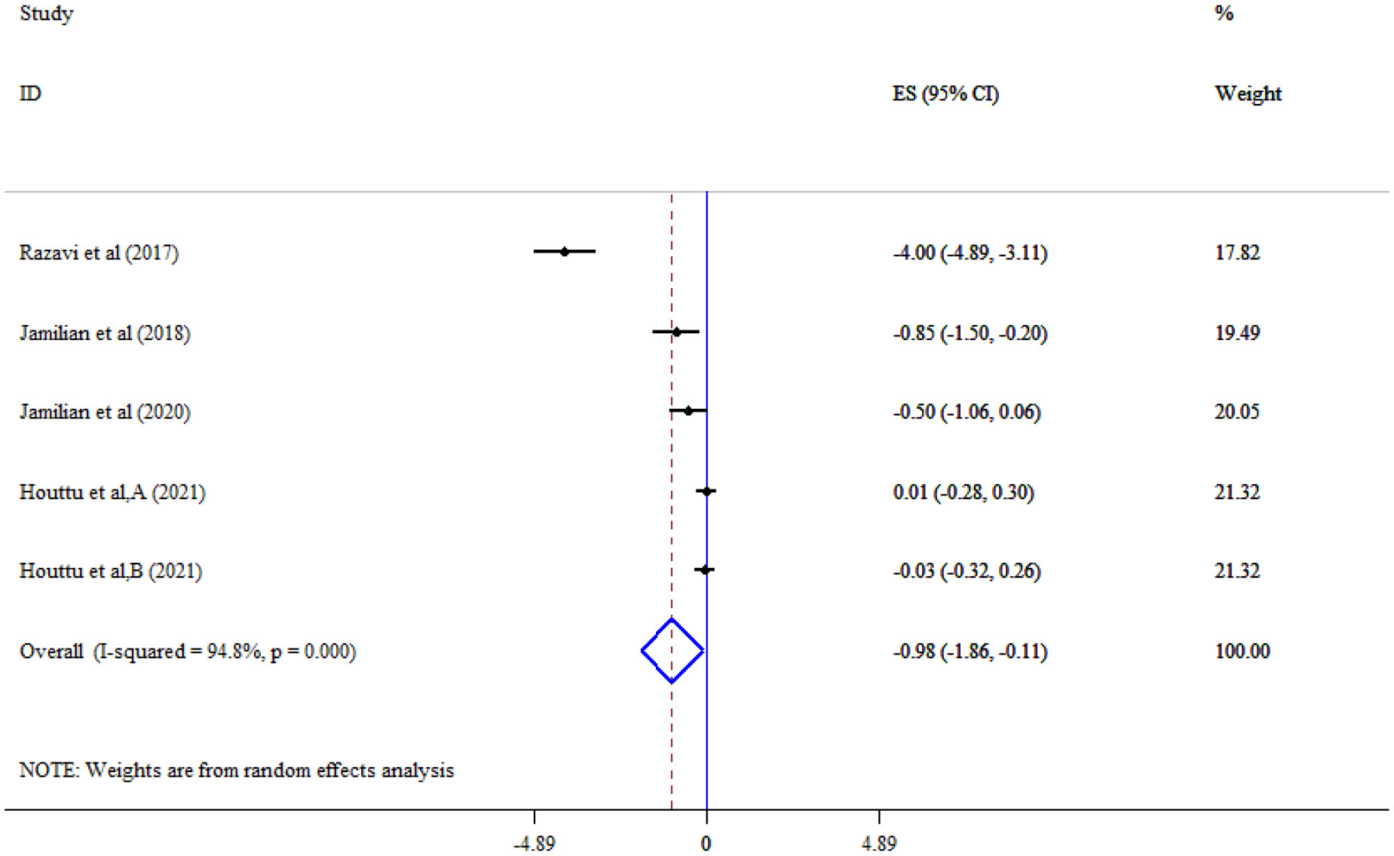
Effect of mega-3 supplementation CRP.

**Figure 9:**
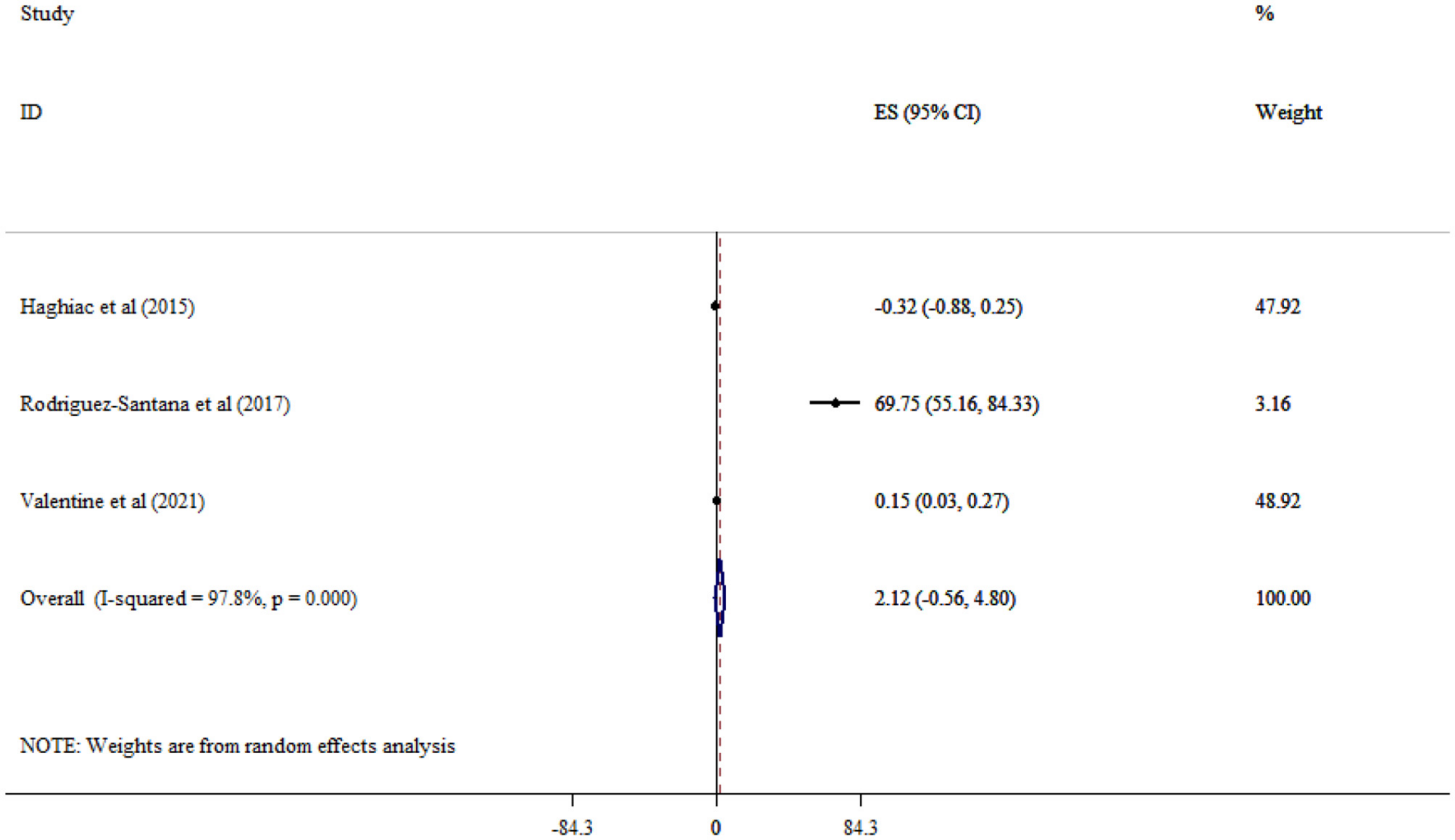
Effect of mega-3 supplementation IL-6.

Omega-3 supplementation significantly reduced CRP (Five studies, SMD = −0.98, 95% CI: −1.86, −0.11; *p* = 0.028; *I*^2^ = 94.8%, *p* < 0.001) (Figure 8), but not IL-6 (Three studies, SMD = 2.12, 95% CI: −0.56, 4.80; *p* = 0.120; *I*^2^ = 97.8%, *p* < 0.001) levels (Figure 9).

### Effect of omega-3 supplementation on oxidative stress

**Figure 10:**
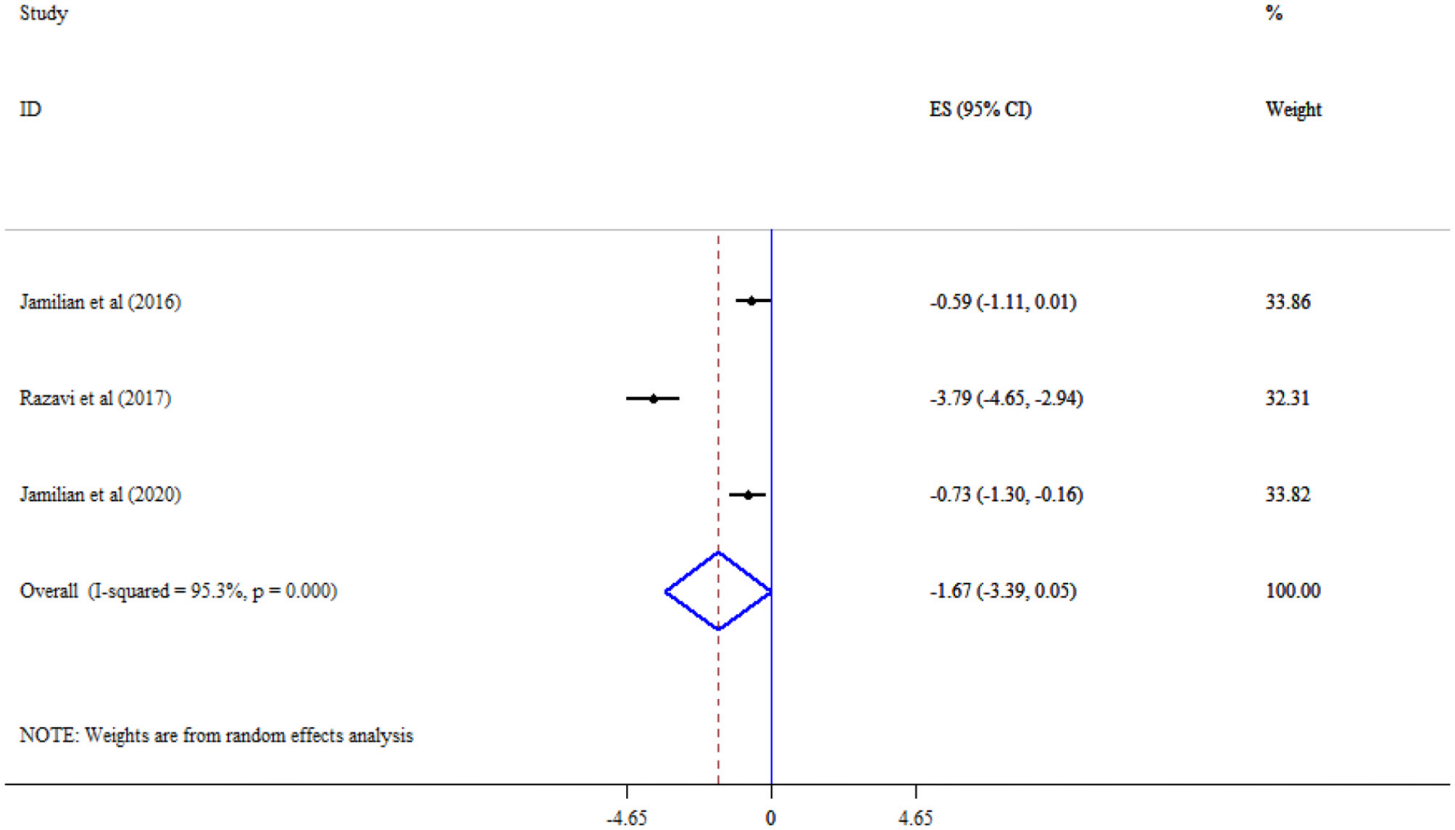
Effect of mega-3 supplementation MDA.

**Figure 11:**
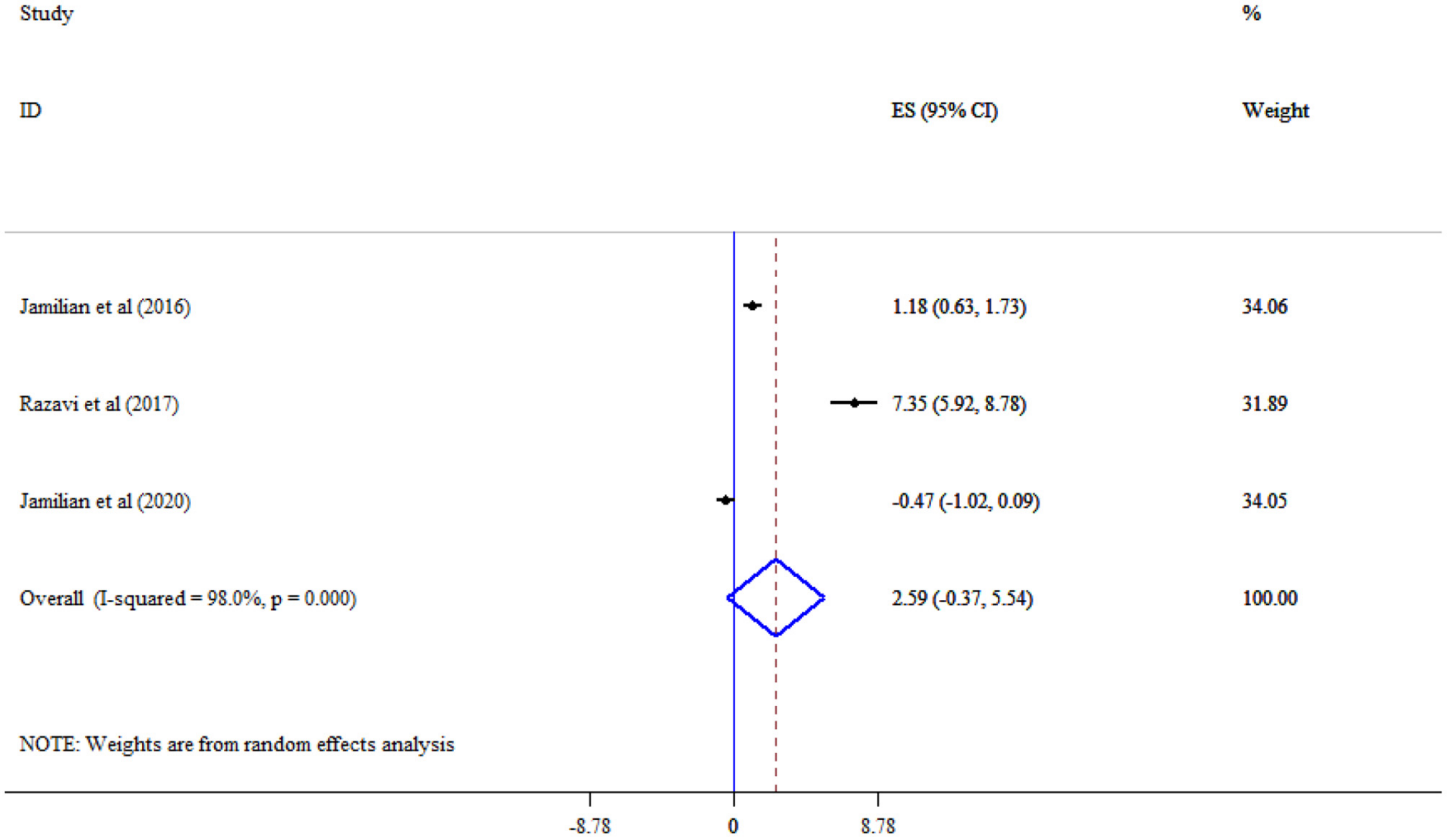
Effect of mega-3 supplementation TAC.

Omega-3 supplementation did not significantly improve MDA (Three studies, SMD = −1.67, 95% CI: −3.39, 0.05; *p* = 0.056; *I*^2^ = 95.3%, *p* < 0.001) (Figure 10), and TAC (Three studies, SMD = 2.59, 95% CI: −0.37, 5.54; *p* = 0.087; *I*^2^ = 98.0%, *p* < 0.001) levels (Figure 11).

### Sensitivity analysis and meta-regression

The overall results were reinforced by sensitivity analysis, which showed significance at *p* < 0.05. Meta-regression analysis demonstrated no linear relationship between effect size and covariates (*p* > 0.05).

## Discussion

This systematic review and meta-analysis evaluated the impact of omega-3 supplementation on maternal glycemic indices, lipid profiles, inflammation, and oxidative stress during pregnancy, drawing on RCTs conducted up to mid-2024. While significant benefits were observed in lipid and inflammatory outcomes, effects on glycemic and oxidative markers were inconclusive. Subgroup analyses were conducted to investigate potential sources of heterogeneity across studies assessing the effects of omega-3 supplementation on FBS, insulin, and TG levels during pregnancy. Notably, the intervention effects appeared to vary according to gestational age at the start of supplementation, intervention duration, sample size, and participants' health conditions.

For FBS, a greater reduction was observed in studies initiating supplementation after 20 weeks of gestation and among women with GDM, while studies involving participants with obesity showed an increase in FBS. Similarly, for insulin, reductions were more prominent in subgroups with later gestational initiation and GDM. A statistically significant decrease in insulin was also found in studies with smaller sample sizes (< 60 participants), where heterogeneity was minimal (*I*^2^ = 6.0%).

Regarding TG, more pronounced reductions were observed in studies with shorter intervention durations (< 15 weeks), larger sample sizes (≥60), and participants with obesity. However, these findings should be interpreted cautiously due to the limited number of studies within several subgroups and the generally high heterogeneity between studies. Overall, these subgroup findings suggest that the effects of omega-3 supplementation during pregnancy may be influenced by maternal health status, timing of intervention, and study design characteristics. While exploratory, these analyses provide insights that may guide future research and targeted clinical applications.

### Glycemic indices

Although our meta-analysis found non-significant trends toward reductions in fasting blood glucose and insulin levels, high heterogeneity suggests that effects may be context-dependent. Previous meta-analyses in gestational diabetes mellitus (GDM) populations have demonstrated modest but statistically significant reductions in fasting plasma glucose and HOMA-IR, along with cuts in hs-CRP (28, 29). However, such effects appear inconsistent across studies, possibly due to differences in baseline insulin resistance, dosage of omega-3, and duration of supplementation.

### Lipid profile

Omega-3 supplementation was associated with a robust reduction in triglycerides and a significant increase in HDL-C, echoing findings from non-pregnant cohorts and pregnant populations. These favorable shifts can be attributed to established mechanisms whereby EPA and DHA inhibit triglyceride synthesis and enhance reverse cholesterol transport (30–32). The lack of significant changes in total and LDL cholesterol may reflect the dual effects of DHA, raising LDL particle size even while modestly affecting LDL levels, as well as variability in dosages and duration across trials.

### Inflammatory markers and oxidative stress

A significant reduction in CRP was observed, consistent with prior evidence in GDM and broader populations. Conversely, IL-6 levels did not change, likely due to limited sample sizes and heterogeneity. Omega-3s may reduce inflammation via suppression of NF-κB signaling, modulation of eicosanoid pathways, and enhanced production of resolvins and protectins (33, 34).

Although our pooled estimates for MDA and TAC were non-significant, individual studies—particularly those combining omega-3 with antioxidant co-supplements—showed improvements in oxidative stress markers. Observational data, such as the TIDES cohort, indicated that third-trimester omega-3 intake was associated with lower maternal urinary markers of oxidative stress (e.g., 8-iso-PGF_2_α decreased by ~10%) (35, 36). The discrepancies may reflect limited statistical power, variability in biomarkers measured, and dosage-dependent antioxidant effects.

### Clinical and research implications

Our findings support the potential role of omega-3 supplementation in improving lipid and inflammatory markers in pregnant women, particularly those with GDM. Given that maternal hypertriglyceridemia and inflammation are risk factors for adverse obstetric outcomes, omega-3 could be a helpful adjunct in clinical nutrition strategies. However, the lack of consistent glycemic and oxidative effects highlights the need for future RCTs with larger sample sizes, standardized dosing (e.g.,≥ 1 g/day EPA + DHA), longer durations, and precise biomarker selection. The combination of omega-3 with antioxidants may further enhance outcomes and warrants investigation.

### Strengths and limitations

The strengths of this meta-analysis include comprehensive literature coverage across multiple databases, rigorous use of Cochrane and PRISMA methodologies, and use of subgroup and meta-regression techniques to explore moderators. Furthermore, due to the limited number of studies, we could not perform subgroup analyses comparing different dosages, duration of supplementation, or timing within pregnancy trimesters.

## Conclusion

Omega-3 PUFA supplementation during pregnancy appears to confer significant reductions in triglyceride and CRP levels and increases in HDL-C, while evidence for glycemic and oxidative outcomes remains inconclusive. Future high-quality, well-powered RCTs are needed to define optimal dosing, timing, and target populations—especially among those with GDM—to elucidate the metabolic benefits of omega-3 in pregnancy fully.

## Data Availability

The datasets presented in this study can be found in online repositories. The names of the repository/repositories and accession number(s) can be found in the article/Supplementary material.
