# Supplementary material for: The effect of omega-3 supplementation on metabolic, inflammatory and oxidative stress biomarkers in pregnant women: a systematic review and meta-analysis
Source: Front Nutr. 2025 Sep 22;12:1639906. doi: 10.3389/fnut.2025.1639906 (PMC12497593; doi:10.3389/fnut.2025.1639906)
Supplement: Supplementary file 1 [file Table_1.DOCX]

Supplementary data

**The search terms for PubMed was as follow:**

(“Omega-3 Fatty Acid”[tiab] OR (Acid AND “Omega-3 Fatty”)[tiab] OR (“Fatty Acid” AND Omega-3)[tiab] OR “Omega 3 Fatty Acid”[tiab] OR “Omega-3 Fatty Acids”[tiab] OR “n-3 Oil”[tiab] OR “Oil AND n-3”[tiab] OR “n 3 Oil”[tiab] OR “n3 Oil”[tiab] OR “Oil AND n3”[tiab] OR “n-3 Fatty Acids”[tiab] OR “n 3 Fatty Acids”[tiab] OR “Omega 3 Fatty Acids”[tiab] OR “n-3 PUFA”[tiab] OR (PUFA AND n-3)[tiab] OR “n 3 PUFA”[tiab] OR “n3 Fatty Acid”[tiab] OR (“Fatty Acid” AND n3)[tiab] OR “n3 PUFA”[tiab] OR (PUFA AND n3)[tiab] OR “n3 Polyunsaturated Fatty Acid”[tiab] OR “n3 Oils”[ti] OR “n-3 Oils”[tiab] OR “n 3 Oils”[tiab] OR “N-3 Fatty Acid”[tiab] OR (Acid AND N-3 Fatty)[tiab] OR (“Fatty Acid” AND N-3)[tiab] OR “N 3 Fatty Acid”[tiab] OR “n-3 Polyunsaturated Fatty Acid”[tiab] OR “n 3 Polyunsaturated Fatty Acid”[tiab]) AND (pregnan*[tiab] OR gestation*[tiab]) AND (lipid*[tiab] OR cholesterol*[tiab] OR Lipoprotein*[tiab] OR “HDL Lipoproteins”[tiab] OR HDL-c[tiab] OR HDL-C[tiab] OR “High-Density Lipoproteins”[tiab] OR “High Density Lipoproteins”[tiab] OR (Lipoproteins AND High-Density)[tiab] OR alpha-Lipoproteins[tiab] OR “alpha Lipoproteins”[tiab] OR “High Density Lipoprotein”[tiab] OR HDL-cholesterol[tiab] OR “High Density Lipoprotein cholesterol”[tiab] OR “alpha-1 Lipoprotein”[tiab] OR “alpha Lipoprotein”[tiab] OR “Low Density Lipoprotein cholesterol”[tiab] OR LDL-C[tiab] OR LDL-c[tiab] OR “Blood Sugar”[tiab] OR (Sugar AND blood)[tiab] OR (Glucose AND blood)[tiab] OR “fasting blood glucose” OR Glucose*[tiab] OR “Glucose Tolerance Tests”[tiab] OR “Oral Glucose Tolerance Test”[tiab] OR OGTT[tiab] OR “Oral Glucose Tolerance”[tiab] OR “Hb A1c”[tiab] OR “Hemoglobin A1c”[tiab] OR (“Hemoglobin A1c” AND Glycosylated)[tiab] OR (“Hemoglobin A1c” AND Glycated)[tiab] OR “Gestational Diabetes”[tiab] OR (“Diabetes Mellitus” AND Gestational)[tiab] OR “Gestational Diabetes Mellitus”[tiab] OR (Diabetes AND Pregnancy-Induced)[tiab] OR GDM[tiab] OR (Birth AND Premature)[tiab] OR (Births AND Premature)[tiab] OR “Premature Births”[tiab] OR “Preterm Birth”[tiab] OR (Birth AND Preterm)[tiab] OR (Births AND Preterm)[tiab] OR “Preterm Births”[tiab] OR inflam*[tiab] OR Cytokin*[tiab] OR Interleukin*[tiab] OR “oxidative stress”[tiab] OR Redox*[tiab] OR “birth weight”[tiab] OR “birth length”[tiab] OR “birth head circumference”[tiab] OR “Birth size”[tiab] OR “Birth sizes”[tiab] OR “neonate weight”[tiab] OR “neonate length”[tiab] OR “neonate head circumference”[tiab] OR “neonate size”[tiab] OR “neonate sizes”[tiab] OR “neonatal weight”[tiab] OR “neonatal length”[tiab] OR “neonatal head circumference”[tiab] OR “neonatal size”[tiab] OR “neonatal sizes”[tiab] OR (Birth[tiab] AND weight[tiab]) OR (Birth[tiab] AND length[tiab]) OR (Birth[tiab] AND “Head Circumference[tiab]) OR (neonate[tiab] AND weight[tiab]) OR (neonate[tiab] AND length[tiab]) OR (neonate[tiab] AND “Head Circumference[tiab]))
